# Melatonin derivative 6a as a PARP-1 inhibitor for the treatment of Parkinson’s disease

**DOI:** 10.3389/fphar.2024.1363212

**Published:** 2024-02-27

**Authors:** Qing-Wei Ma, Rui-Ting Han, Zi-Jie Wu, Jun-Jie Zhou, Meng-Ting Chen, Xiang-Zhi Zhang, Wen-Zhe Ma, Na Feng

**Affiliations:** ^1^ School of Pharmacy and Food Engineering, Wuyi University, Jiangmen, China; ^2^ State Key Laboratory of Quality Research in Chinese Medicine, Macau University of Science and Technology, Macao, China

**Keywords:** melatonin derivative, Parkinson’s disease, PARP-1, mTOR, mitochondrial dysfunction

## Abstract

Both continuous oxidative stress and poly (ADP-ribose) polymerase 1 (PARP-1) activation occur in neurodegenerative diseases such as Parkinson’s disease. PARP-1 inhibition can reverse mitochondrial damage and has a neuroprotective effect. In a previous study, we synthesized melatonin derivative 6a (MD6a) and reported that it has excellent antioxidant activity and significantly reduces α-synuclein aggregation in *Caenorhabditis elegans*; however, the underlying mechanism is largely unknown. In the present study, we revealed that MD6a is a potential PARP-1 inhibitor, leading to mammalian targe of rapamycin/heat shock factor 1 signaling downregulation and reducing heat shock protein 4 and 6 expression, thus helping to maintain protein homeostasis and improve mitochondrial function. Together, these findings suggest that MD6a might be a viable candidate for the prevention and treatment of Parkinson’s disease.

## 1 Introduction

Parkinson’s disease (PD) is a common neurological degenerative disease in middle-aged and older adults; most patients develop the disease after the age of 60. Its clinical manifestations are motor dysfunction, reduced cognitive function, and depression (Jankovic, 2007), which can seriously affect patients’ quality of life. In recent years, mitochondrial dysfunction has been considered as a crucial defect occurring in the early PD pathogenesis for the loss of dopaminergic neurons (Subramaniam and Chesselet, 2013), which is closely related to aging (Chandra et al., 2017), environmental exposure (Huang et al., 2022), and genetic factors (Ye et al., 2023). Mitochondrial dysfunction and damage—including mitochondrial DNA mutations, mitochondrial electron transport chain (ETC) dysfunction, and mitochondrial reactive oxygen species (ROS) production increase—are widespread in patients with PD. This dysfunction leads to reduced energy production in the mitochondria and increased oxidative stress, which can subsequently cause cell damage and neuronal degeneration (Prasuhn et al., 2020). Studies have shown that more than 90% of sporadic PD cases are caused by mitochondrial dysfunction, which compromise the power source of nerve cells. This aggravates the accumulation of damaged mitochondria, which fails to produce enough energy for the cell, and causes the gradual death of neurons, and eventually leading to the development of PD (Magalhaes et al., 2021).

Poly (ADP-ribose) polymerase 1 (PARP-1) is a DNA repair enzyme that is mainly found in the nuclei of eukaryotes (Bai, 2015; Ray Chaudhuri and Nussenzweig, 2017). Under normal physiological conditions, PARP-1 is as a sensor of unligated Okazaki fragments during DNA replication and assists their repair (Hanzlikova et al., 2018). In pathological states, a large amount of DNA is damaged and PARP-1 becomes overactivated. This inhibits mitochondrial ETC activity, thus resulting in mitochondrial energy metabolism disorders, chromatin agglutination, and eventually programmed cell death activated by PARP-1 (known as parthanatos) (Wang et al., 2009; Wang et al., 2011; Wang et al., 2016). Continuous oxidative stress and PARP-1 activation are present in neurodegenerative diseases such as PD, and PARP-1-mediated parthanatos is one of the main forms of neuronal death in this disease (Martire et al., 2015). Inhibiting PARP-1 activation can reverse mitochondrial damage and reduce genetic defects of mitochondrial metabolism, which has a neuroprotective effect (Sun et al., 2021).

Melatonin is a natural antioxidant in the human body. A relatively high concentration of melatonin is maintained in neuronal mitochondria (Reiter et al., 2017); this mitochondria-targeted melatonin removes free radicals generated by oxidative phosphorylation via various antioxidant pathways, protects mitochondrial complexes I and IV, and increases adenosine triphosphate (ATP) synthesis (Díaz-Casado et al., 2017). Moreover, studies have reported that melatonin has a neuroprotective effect in PD. Melatonin can reduce nitrite release from astrocytes (Gonzalez, 2020), decrease the neurotoxic effects of nitric oxide (López et al., 2017), reduce dopaminergic neuron apoptosis, and prevent neuroinflammation. It can also inhibit the formation of Lewy bodies by affecting the expression and aggregation of α-synuclein (α-syn), or just directly binding to α-syn, thus improving the dopaminergic system (Sae-Ung et al., 2011).

In a previous study, we synthesized the C7-substituted melatonin derivative 6a (MD6a) using a C/H functionalization reaction. We revealed that MD6a has good antioxidant activity and can reduce ROS levels in wild-type N2 and NL5901, with an optimal concentration of 10 μM (He et al., 2022). We utilized *C. elegans* (*Caenorhabditis elegans*) as a model studying for the PD pathology because it has several advantages, including a short life cycle, easy to maintain and eight DAergic neurons containing conserved DAergic pathway and genes with human orthologs. Moreover, its PD-like phenotypes can be easily generated and analyzed in laboratory (Brunetti et al., 2020; Caldwell et al., 2020). We found that MD6a significantly reduces α-syn aggregation in NL5901 nematodes and has neuroprotective effects against 6-hydroxydopamine (6-OHDA)-induced dopaminergic neuron damage. These results suggest that MD6a may be a potential treatment for PD. However, the mechanism and targets underlying the neuroprotective effects of MD6a in PD remain largely unknown. In this study, we demonstrated that MD6a acts as a PARP-1 inhibitor to reduce α-syn aggregation and enhance mitochondrial function in a *C. elegans* PD model.

## 2 Materials and methods

### 2.1 Strains and maintenance

The wild type worm (Bristol N2) and transgenic line of NL5901 (pkIs2386, unc-54p::alphasynuclein::YFP) were obtained from the *Caenorhabditis* Genetics Center (CGC). Worms were grown on the nematode growth medium (NGM) agar plate and fed with *Escherichia coli* OP50 at 20°C (Brenner, 1974). Synchronization of nematode culture was achieved using treatment with sodium hypochlorite and 1 M NaOH (1:1). The synchronized eggs were cultured in M9 buffer for 24 h at 20°C to hatch L1 larvae. Then, the L1 stage worms were transferred to NGM plate.

### 2.2 RNAi

RNAi feeding experiments were performed on synchronized L1 to L4 larvae at 20°C. *E. coli* HT115 (DE3) containing empty vector (pL4440) or target genes strains were cultured overnight in LB medium containing 100 μg/mL ampicillin and 12.5 μg/mL tetracycline at 37°C. Then spread to NGM plates containing 25 μg/mL carbenicillin and 1 mM isopropyl 1-thio-b-β-galactopyranoside (IPTG) at 37°C overnight. The synchronized NL5901 worms were transferred to NGM plates seeded with RNAi bacteria and allowed to grow until mature. RNAi efficiency was evaluated prior to starting the experiment.

### 2.3 Analysis of α-syn aggregation

Effect of MD6a on the α-syn aggregation was evaluated using NL5901 strain. Briefly, age-synchronized worms were washed three times with M9 buffer to get rid of remaining bacteria and mounted onto 2% agarose pads. Then, worms were immobilized with 20 mM sodium azide. To monitor the α-syn aggregation, YFP protein was visualized and photographed with a fluorescence microscope (Olympus BX63). The fluorescence intensity was quantified using ImageJ.

### 2.4 ATP level analysis

Briefly, the collected worms added extract liquid were grinded by freeze-thawing with liquid nitrogen. Centrifugation was performed at 4°C for 10 min at 12,000 rmp and 30 µL of the supernatant was taken and then assayed with the ATP Content Assay Kit (Solarbio). And protein quantification was performed with BCA Protein Assay kit (Biosharp).

### 2.5 OCR assay

Oxygen consumption rates were measured using the Oxytherm (Hansatech, United Kingdom), a Clark-type oxygen electrode as described previously (Schulz et al., 2007). The collected nematodes were resuspended in 1 mL of M9 and transferred to the chamber. Oxygen concentration was monitored with a Clark electrode in a closed chamber for 10 min. The nematodes were subsequently collected. Protein concentration was measured using the BCA Protein Assay kit.

### 2.6 Swimming assay

At least 15 worms on day 0 of adulthood were randomly selected and transferred to a glass slide containing 10 µL M9 buffer. After allowing the worms to acclimate to the liquid medium for 30 s, their movement was continuously recorded by a microscope (MZ62) for 10 s.

### 2.7 Mitochondrial ROS assay

To determine mitochondrial ROS levels, nematodes were stained with MitoSOX™ Red for 20 min. Then, the worms were anaesthetised with 20 mM sodium azide solution and mounted on microscopic slides. The fluorescence was examined under a fluorescence microscope (Olympus BX63). The Fluorescence intensity was quantified using ImageJ.

### 2.8 Measurement of mitochondrial membrane potential

Nematodes were cultured to the L3 stage placed into 100 nM TMRE dishes stained for 24 h, and worms were transferred to plates without dye for 1 h prior to imaging to clear the gut of residual dye (Berry et al., 2023). Nematodes were mounted on 2% agarose pads under 20 mM sodium azide anesthesia. Changes in mitochondrial membrane potential fluorescence intensity were observed using a confocal (Leica TCS SP8). Fluorescence intensity was quantified using ImageJ.

### 2.9 Quantitative real-time polymerase chain reaction (qPCR) analysis

Nematodes were treated with MD6a to incubate the L4 stage, and total RNA was extracted from nematodes of each treatment group using TRIzol reagent (Invitrogen), as previously described (Lapierre et al., 2013). The RNA of worms was reverse transcribed into cDNA by using PrimeScript™ RT reagent Kit with gDNA Eraser. Real-time PCR was performed according to the primers designed in Table 1.

**TABLE 1:** Primers used for qPCR.

Gene	Primer sequences
β-Actin-F	GTC​GGA​AGA​CCA​CGT​CAT​C
β-Actin-R	CAC​GAA​GCT​CAT​TGT​AGA​AGG
*parp-1*-F	CTTGTCAAGCTGCCCATT
*parp-1*-R	CGC​TGA​TTT​GAT​CAT​ACG​CG
*let-363*-F	CGA​TGG​ACG​AAC​AGA​TAT​AGC​CTC
*let-363*-R	TCG​CAA​TCA​GAA​AAG​CGA​GAG​C
*hsf-1*-F	GGT​GGT​CTA​ACT​CGA​ACA​GA
*hsf-1*-R	CAC​GCA​TCT​CTG​CCA​TTA​C
*hsp-4*-F	GTG​CGT​TGG​AGT​CTT​CAA​GA
*hsp-4*-R	CCAGTGCTTGATGTCTTG
*hsp-6*-F	CTCGCCTATGGATTGGAT
*hsp-6*-R	GAT​CAA​CTC​CTT​GCT​CCT​TC

## 3 Results

### 3.1 MD6a acts as a PARP-1 inhibitor to reduce α-syn aggregation in *Caenorhabditis elegans*

**FIGURE 1:**
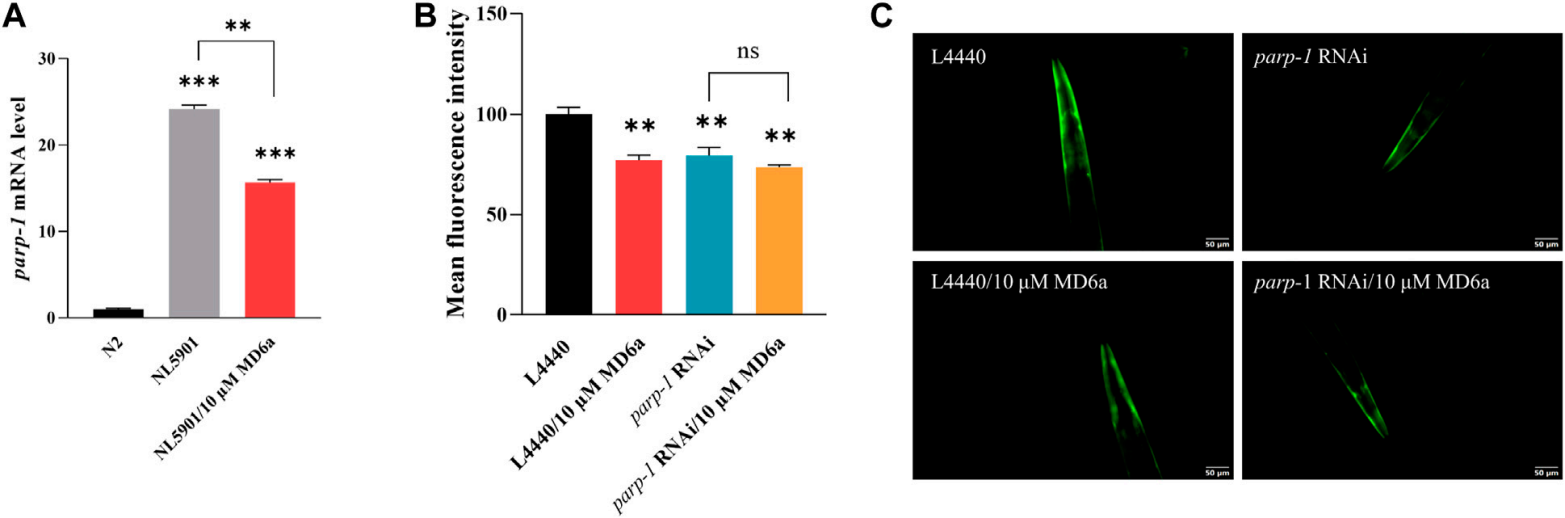
The MD6a-mediated reduction of α-syn in *Caenorhabditis elegans* is dependent on *parp-1*. **(A)** qPCR measurement of *parp-1* expression in N2, NL5901 and 10 µM MD6a-treated NL5901 worms. **(B, C)** Quantification **(B)** and fluorescence images **(C)** of α-syn levels in NL5901 worms treated with 10 μM MD6a and *parp-1* RNAi. Differences were analyzed using the *t*-test; *p < 0.05, **p < 0.01, NS (no significance), compared with the control group.

The accumulation and aggregation of α-syn is known as one of the pathological features in PD patients. Previous studies have demonstrated that pathological α-syn can activate PARP-1, thus leading to the loss of dopaminergic neurons in PD (Kam et al., 2018). We used the *C. elegans* strain NL590, which expresses human α-syn fused with YFP under an *unc-54* promoter, to elucidate the effects of MD6a on α-syn aggregation. The *parp-1* mRNA levels in wild-type N2 and NL5901 worms were examined by qPCR. The result showed *parp-1* expression in NL5901 nematodes was significantly higher than that in N2 worms, treatment with 10 μM MD6a significantly reduced *parp-1* mRNA levels in NL5901 nematodes (Figure 1A). To confirm the role of *parp-1* in the neuroprotective effects of MD6a, we genetically knocked down *parp-1* expression using RNAi. Reduced α-syn accumulation was observed in NL5901 worms treated with both 10 μM MD6a and *parp-1* RNAi (Figures 1B,C). However, under *parp-1* RNAi, 10 μM MD6a failed to further reduce α-syn aggregation in NL5901 nematodes compared with *parp-1* RNAi group (Figures 1B,C). Together, these results indicate that the MD6a-induced reduction of α-syn aggregation in *C. elegans* is dependent on *parp-1*.

### 3.2 MD6a enhances mitochondrial function in a PD model through *parp-1*

**FIGURE 2:**
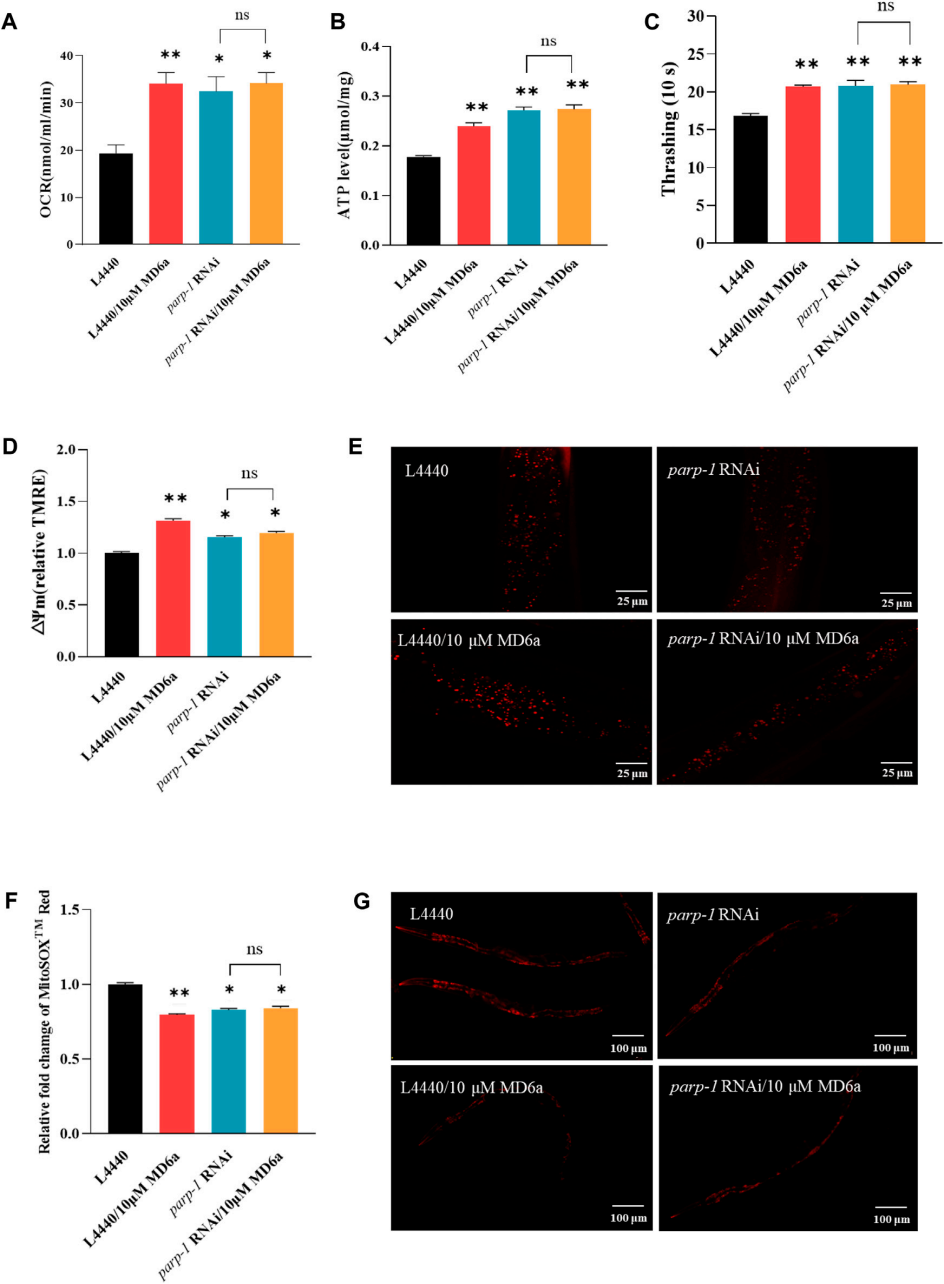
MD6a-mediated protection of mitochondrial function in *Caenorhabditis elegans* is dependent on *parp-1*. **(A)** Rate of oxygen consumption in NL5901 worms treated with 10 μM MD6a and *parp-1* RNAi. **(B)** ATP levels in NL5901 worms treated with 10 μM MD6a and *parp-1* RNAi. **(C)** Movement ability in NL5901 worms treated with 10 μM MD6a and *parp-1* RNAi. **(D, E)** Quantification **(D)** and fluorescence images **(E)** of mitochondrial membrane potential levels in NL5901 worms treated with 10 μM MD6a and *parp-1* RNAi. **(F, G)** Quantification **(F)** and fluorescence images **(G)** of mitochondrial ROS levels in NL5901 worms treated with 10 μM MD6a and *parp-1* RNAi. Differences were analyzed using the *t*-test; *p < 0.05, **p < 0.01, NS (no significance), compared with the control group.

Mitochondrial dysfunction is widely considered as a main contributor to neurodegeneration in PD (Park et al., 2018). The complex interplay between mitochondrial dynamics and bioenergetics is especially important for neuronal function. In general, neurons have high energy demands that require many functional mitochondria. The activation of pathological *parp-1* causes mitochondrial dysfunction, leading to ATP depletion and mitochondrial membrane potential decline (Cipriani et al., 2005; Luo and Kraus, 2011). To elucidate whether MD6a acts as a PARP-1 inhibitor to contribute to protecting mitochondrial function in PD, we measured the oxygen consumption rate (OCR), ATP levels, movement ability, mitochondrial ROS production and mitochondrial membrane potential. The results demonstrated that MD6a treatment significantly affected mitochondrial biogenesis in NL5901 worms, with markedly increased OCR levels (Figure 2A), ATP production (Figure 2B), movement ability (Figure 2C) and mitochondrial membrane potential (Figures 2D,E), while observably decreased mitochondrial ROS levels (Figures 2F,G). Furthermore, *parp-1* RNAi could mimic all these beneficial effects of MD6a which suggested that MD6a improve mitochondrial dysfunction through *parp-1*. However, MD6a could not further increased OCR, ATP, movement, mitochondrial membrane potential levels (Figures 2A–E), and decreased mitochondrial ROS levels (Figures 2F,G) under *parp-1* RNAi. Taken together, these results indicate that MD6a significantly enhances mitochondrial function in the PD nematode model through *parp-1*.

### 3.3 MD6a inhibits PARP-1 to reduce α-syn aggregation through *let-363* in *Caenorhabditis elegans*

**FIGURE 3:**
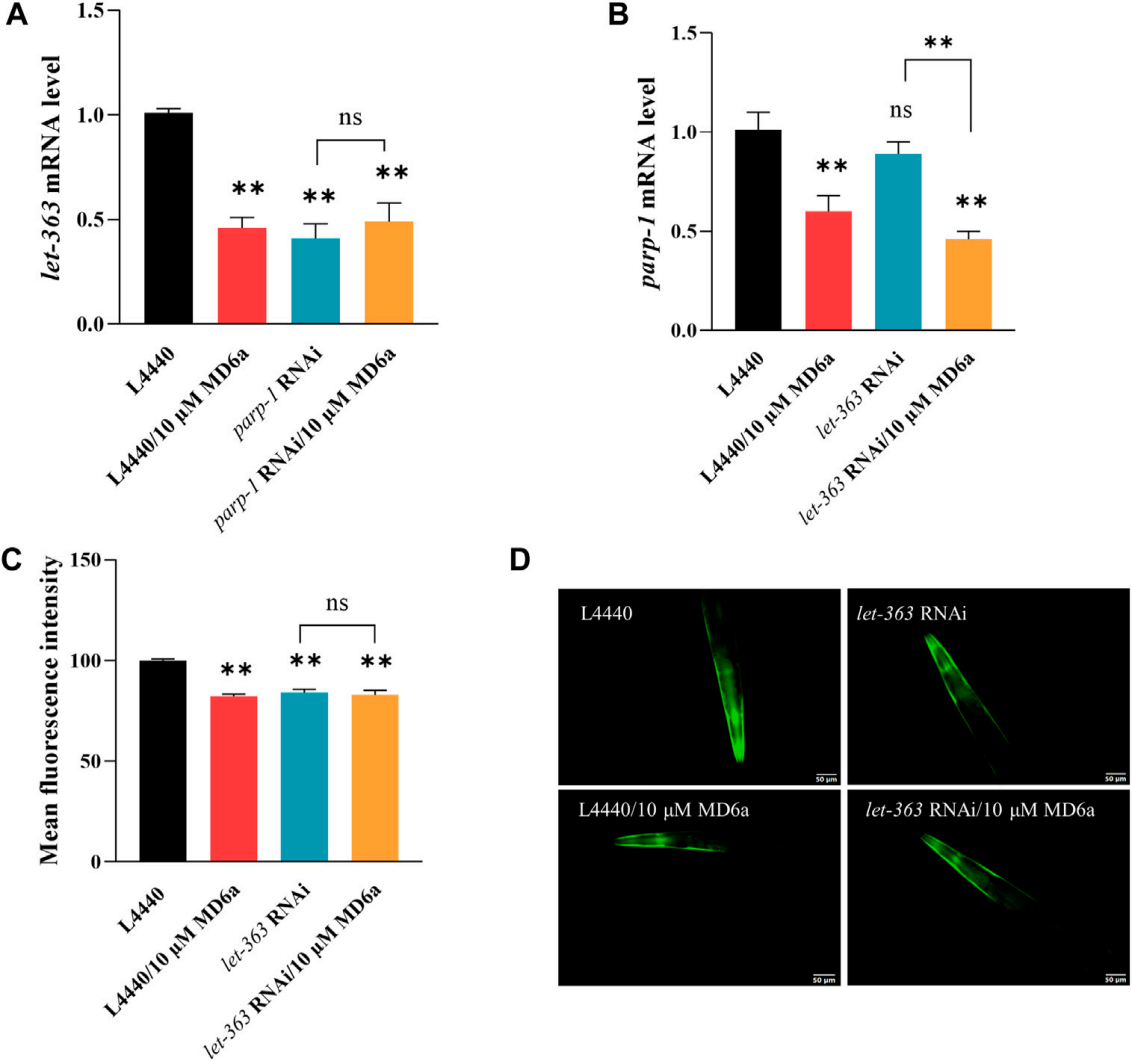
MD6a affects *let-363* to exert neuroprotective effects in PD. **(A)** qPCR measurement of *let-363* expression in NL5901 worms treated with 10 µM MD6a and *parp-1* RNAi. **(B)** qPCR measurement of *parp-1* expression in NL5901 worms treated with 10 µM MD6a and *let-363* RNAi. **(C, D)** Quantification **(C)** and fluorescence images **(D)** of α-syn levels in NL5901 worms treated with 10 μM MD6a and *let-363* RNAi. Results are presented as the mean ± SEM of three independent experiments performed in triplicate. Differences were analyzed using the *t*-test; *p < 0.05, **p < 0.01, NS (no significance), compared with the control group.

The mammalian target of rapamycin (mTOR) signaling pathway plays an important homeostatic function in the regulation of energy metabolism, cell survival, senescence, and neurodegeneration (Wullschleger et al., 2006). Moreover, increasing evidence indicates that mTOR is critical for the pathogenesis of PD. A previous study reported that mTOR protein expression levels are increased in the temporal cortex of patients with α-syn accumulation (Crews et al., 2010). We explored the effects of MD6a on the TOR signaling pathway in *C. elegans*, and revealed that the expression of *let-363*, an ortholog of TORC1, was downregulated by 10 μM MD6a (Figure 3A). Furthermore, although 10 μM MD6a did not decrease *let-363* expression in *parp-1* RNAi-treated worms (Figure 3A), it decreased *parp-1* expression in *let-363* RNAi-treated worms (Figure 3B). These results suggest that the MD6a-mediated regulation of *let-363* is dependent on *parp-1*. Additionally, the fluorescence intensity of α-syn was reduced by *let-363* RNAi treatment in NL5901 nematodes; however, 10 μM MD6a did not reduce α-syn fluorescence intensity in *let-363* RNAi-treated nematodes compared with *let-363* RNAi group (Figure 3D). These findings indicate that MD6a downregulates *let-363* in a *parp-1*-dependent manner to reduce α-syn aggregation.

### 3.4 MD6a inhibits PARP-1 via *let-363/hsf-1* in *Caenorhabditis elegans*

**FIGURE 4:**
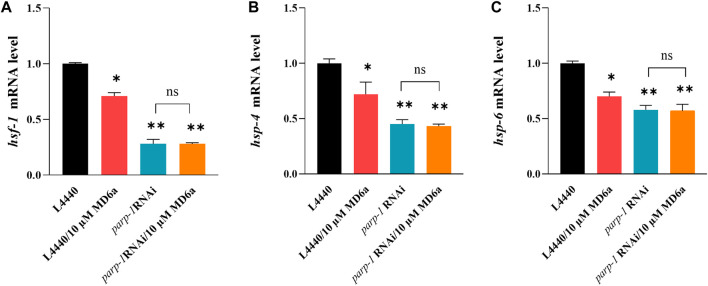
MD6a mediates *hsf-1* to exert neuroprotective effects in PD. **(A)** qPCR measurement of *hsf-1* expression in NL5901 worms treated with 10 µM MD6a and *parp-1* RNAi. **(B)** qPCR measurement of *hsp-4* expression in NL5901 worms treated with 10 µM MD6a and *parp-1* RNAi. **(C)** qPCR measurement of *hsp-6* expression in NL5901 worms treated with 10 µM MD6a and *parp-1* RNAi. Results are presented as the mean ± SEM of three independent experiments performed in triplicate. Differences were analyzed using the *t*-test; *p < 0.05, **p < 0.01, NS (no significance), compared with the control group.

Previous studies have identified a number of transcription factors downstream of TOR, including DAF-16/FOXO, SKN-1/NRF, HSF-1/HSFs, PHA-4/FOXA, HLH-30/TFEB, and RPC-1/POL III (Blackwell et al., 2019). In this study, we found *hsf-1* mRNA levels were downregulated by treatment with 10 μM MD6a, and *parp-1* RNAi blocked this MD6a-mediated reduction (Figure 4A). Previous reports have demonstrated that α-syn aggregation is related to dysfunction of the protein degradation pathway, including of heat shock proteins (HSPs) (Jones et al., 2014). HSP-4 and HSP-6 are involved in regulating intracellular protein degradation processes by binding to damaged or obsolete proteins and removing unstable proteins via endoplasmic reticulum-associated protein degradation (ERAD) (Shin et al., 2022) and mitochondria-associated degradation pathway (Rolland et al., 2019). We thus evaluated the mRNA levels of *hsp-4* and *hsp-6*, which are associated with HSPs in NL5901 worms. The results revealed that MD6a significantly reduced degradation-regulated genes in NL5901 nematodes (Figures 4B,C). Furthermore, *parp-1* RNAi reduced the expression levels of *hsp-4* and *hsp-6* in NL5901 worms. However, 10 μM MD6a treatment could not further reduce the mRNA levels of *hsp-4* and *hsp-6* under *parp-1* RNAi (Figures 4B,C). Together, these results indicate that MD6a inhibits PARP-1 downregulation of the *let-363*/*hsf-1* signaling pathway by targeting the reduction of *hsp-4* and *hsp-6* mRNA levels.

## 4 Discussion

HSF-1 is a heat shock transcription factor that regulates the synthesis of heat shock proteins to assist cells cope with proteotoxic stress (Kyriakou et al., 2022). It is also a key effector of longevity signaling (Lu et al., 2020). Recent studies have reported that HSF-1 plays crucial role in the pathogenesis of PD (Govindan et al., 2018; Zheng et al., 2023). Hyperactivation of HSF-1 was associated with the onset of PD, and the elevated activity of HSF-1 and increased expression levels of heat shock proteins in patients with PD lead to increased sensitivity of neurons to proteotoxic stress, which accelerate the progression of the disease (Kim et al., 2016). Although the exact relationship between HSF-1 and PD remains to be elucidated in further studies, the current findings suggest that HSF-1 may play an important role in the pathogenesis and progression of PD by regulating the synthesis of heat shock proteins, which affect neuronal survival and function.

Mitochondrial dysfunction and protein homeostasis imbalances are two essential factors in the pathogenesis of Parkinson’s, which are closely linked (Chiti and Dobson, 2017; Malpartida et al., 2020). It has been found that misfolded proteins in the cytoplasm are recruited to mitochondria via chaperone proteins and degraded by mitochondrial proteases, which facilitate the cell maintain protein homeostasis (Li et al., 2019). However, excessive accumulation of mitochondria unfolded proteins response (UPR^MT^) can compromise mitochondrial integrity and accelerate the symptom of PD. In *C. elegans*, α-syn and PD-associated disease variants can not only induce the UPR^MT^, but also dysregulate the UPR^MT^ synergistically potentiate dopaminergic neurotoxicity (Martinez et al., 2017). Various studies revealed that persistent endoplasmic reticulum (ER) stress has been linked to neurodegenerative diseases, such as PD. Anesthesia-induced neurotoxicity is related to ER stress, which is attenuated by HSP-4 downregulation (Shin et al., 2022). HSF-1 decreases the expression of HSP-4 and HSP-6, which contributes to the maintenance of ER and mitochondrial homeostasis, reduces ER stress and mitochondrial damage, and thus protects neurological function.

## 5 Conclusion

**FIGURE 5:**
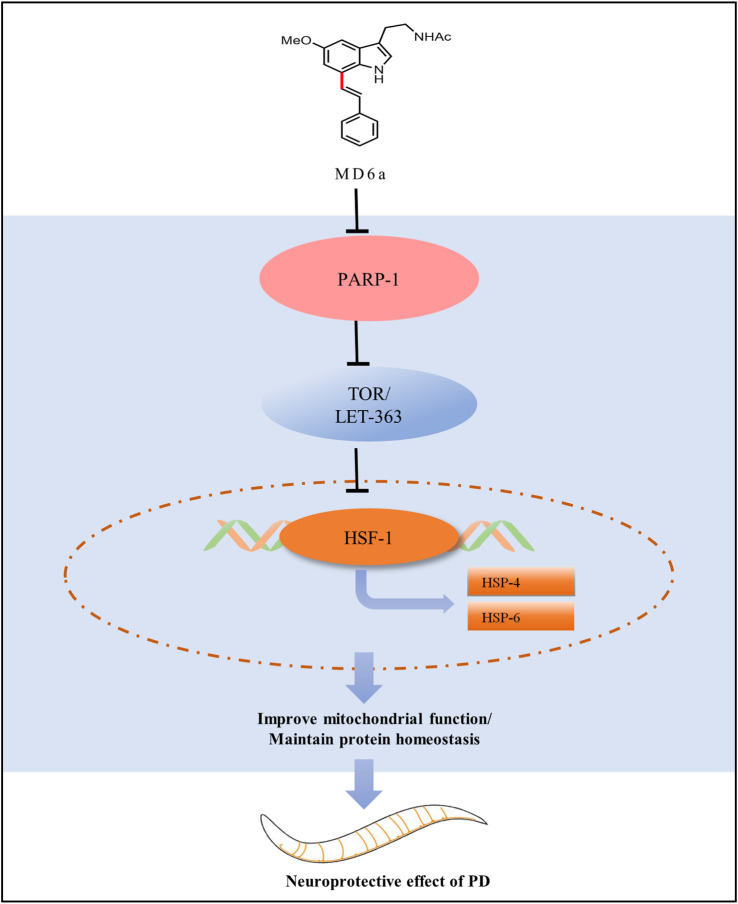
Working model for the complete pathway through which MD6a may exert a neuroprotective role in PD.

In the present study, we found that MD6a acts as a PARP-1 inhibitor to reduce α-syn aggregation and enhance mitochondrial function in *C. elegans* through TOR/HSF-1 signaling. MD6a inhibits PARP-1 to downregulate *let-363*/*hsf-1* signaling by targeting *hsp-4* and *hsp-6*, thus improving mitochondrial function and maintaining protein homeostasis in PD (Figure 5). Together, our findings indicate that MD6a may serve as a potential PARP-1 inhibitor for the prevention and treatment of PD.

## Data Availability

The original contributions presented in the study are included in the article/Supplementary material, further inquiries can be directed to the corresponding author.
